# Bis(2,2′-bipyrid­yl)bromidocopper(II) bromide bromo­acetic acid hemihydrate

**DOI:** 10.1107/S1600536809048995

**Published:** 2009-11-21

**Authors:** Yaru Liu, Junshan Sun, Xinli Wang

**Affiliations:** a School of Science, North University of China, Taiyuan 030051, Shanxi, People’s Republic of China; b Department of Materials and Chemical Engineering, Taishan University, 271021 Tai’an, Shandong, People’s Republic of China; c Department of State-owned Assets, Taishan University, 271021 Tai’an, Shandong, People’s Republic of China

## Abstract

In the title compound, [CuBr(C_10_H_8_N_2_)_2_]Br·BrCH_2_COOH·0.5H_2_O, the Cu^II^ ion is coordinated by four N atoms [Cu—N = 1.985 (6)–2.125 (7) Å] from two 2,2′-bipyridine ligand mol­ecules and a bromide anion [Cu—Br = 2.471 (2) Å] in a distorted trigonal-bipyramidal geometry. Short centroid–centroid distances [3.762 (5) and 3.867 (5) Å] between the aromatic rings of neighbouring cations suggest the existence of π–π inter­actions. Inter­molecular O—H⋯Br hydrogen bonds and weak C—H⋯O and C—H⋯Br inter­actions consolidate the crystal packing.

## Related literature

For related structures, see: Hammond *et al.* (1999 ▶); Song *et al.* (2004 ▶).
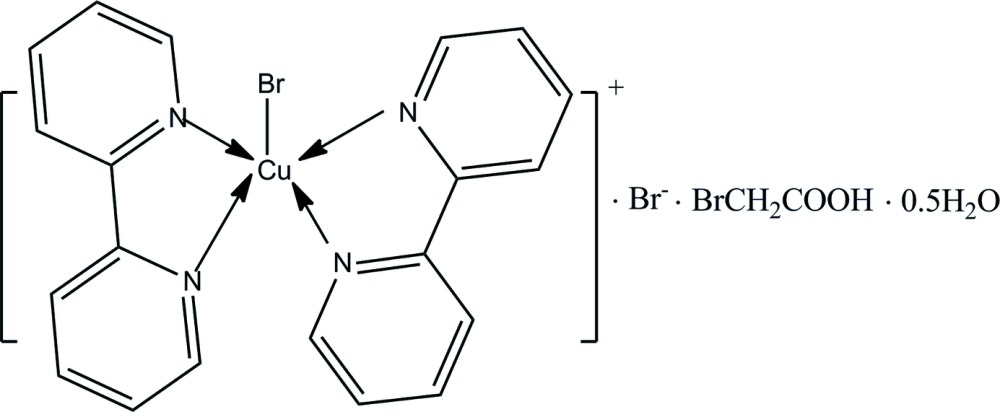


## Experimental

### 

#### Crystal data

[CuBr(C_10_H_8_N_2_)_2_]Br·C_2_H_3_BrO_2_·0.5H_2_O
*M* *_r_* = 683.69
Triclinic, 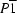

*a* = 8.580 (4) Å
*b* = 12.125 (6) Å
*c* = 13.212 (6) Å
α = 70.295 (9)°
β = 81.427 (8)°
γ = 76.669 (9)°
*V* = 1255.3 (11) Å^3^
*Z* = 2
Mo *K*α radiation
μ = 5.67 mm^−1^
*T* = 273 K
0.30 × 0.27 × 0.26 mm

#### Data collection

Bruker or SMART APEX diffractometer
Absorption correction: multi-scan (*SADABS*; Bruker, 2005 ▶) *T*_min_ = 0.281, *T*_max_ = 0.320
6360 measured reflections
4384 independent reflections
2689 reflections with *I* > 2σ(*I*)
*R*_int_ = 0.113

#### Refinement

*R*[*F* ^2^ > 2σ(*F* ^2^)] = 0.067
*wR*(*F* ^2^) = 0.183
*S* = 1.01
4384 reflections
299 parameters
3 restraints
H-atom parameters constrained
Δρ_max_ = 1.20 e Å^−3^
Δρ_min_ = −1.10 e Å^−3^

### 

Data collection: *SMART* (Bruker, 2005 ▶); cell refinement: *SAINT* (Bruker, 2005 ▶); data reduction: *SAINT*; program(s) used to solve structure: *SHELXS97* (Sheldrick, 2008 ▶); program(s) used to refine structure: *SHELXL97* (Sheldrick, 2008 ▶); molecular graphics: *XP* in *SHELXTL* (Sheldrick, 2008 ▶); software used to prepare material for publication: *SHELXL97*.

## Supplementary Material

Crystal structure: contains datablocks I, global. DOI: 10.1107/S1600536809048995/cv2644sup1.cif

Structure factors: contains datablocks I. DOI: 10.1107/S1600536809048995/cv2644Isup2.hkl

Additional supplementary materials:  crystallographic information; 3D view; checkCIF report

## Figures and Tables

**Table 1:** Hydrogen-bond geometry (Å, °)

*D*—H⋯*A*	*D*—H	H⋯*A*	*D*⋯*A*	*D*—H⋯*A*
O1—H1⋯Br3	0.82	2.34	3.129 (7)	163
O3—H3*A*⋯Br3	0.85	2.64	3.426 (11)	154
C14—H14⋯O1^i^	0.93	2.46	3.254 (12)	144
C3—H3⋯O2^ii^	0.93	2.60	3.374 (12)	141
C2—H2*B*⋯Br2^iii^	0.97	2.88	3.815 (11)	163
C13—H13⋯Br1^iv^	0.93	2.89	3.750 (10)	154

Symmetry codes: (i) 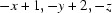; (ii) 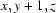; (iii) 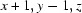; (iv) 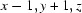.

**Fig. 1.**
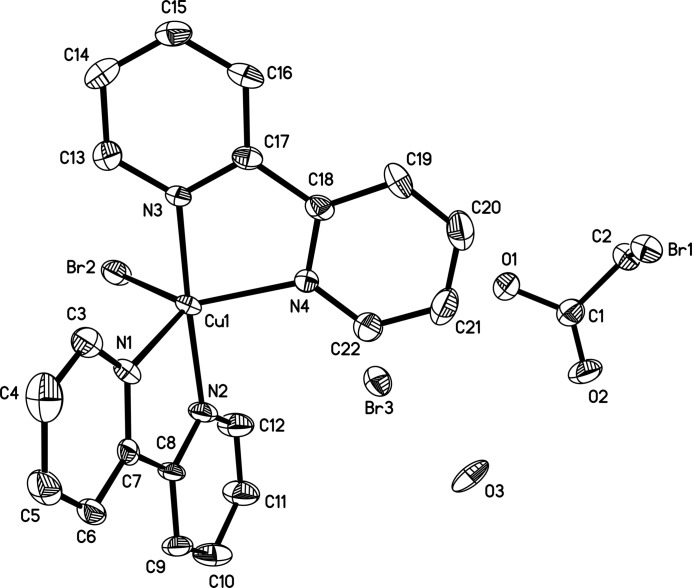
The molecular structure of (I) showing the atomic numbering and 30% probability displacement ellipsoids. H atoms omitted for clarity.

## supplementary crystallographic information

### Comment

Metal complexes with carboxylates are among the most investigated complexes in the field of coordination chemistry. In addition, metal-2,2'-bipyridine complexes and their derivatives have attracted much attention during recent decades because of their structural features (Hammond *et al.*, 1999; Song *et al.*, 2004). In this work, we present the crystal structure of the title compound (I) obtained from the bromoacetic acid and cupric acetate in the presence of co-ligand of 2,2'-bipyridine.

In (I) (Fig.1), the Cu^II^ ion exhibits a five-coordinated trigonal bipyramidal geometry with four N atoms [Cu—N 1.985 (6)–2.125 (7) Å] from two 2,2'-bipyridine ligand molecules and a Br anion [Cu—Br 2.471 (2) Å]. Two N atoms and coordinated Br anion form an equatorial plane. The two rest coordinated N atoms occupy the apical positions with the N—Cu—N angle of 175.6 (3)/%. Intermolecular O—H···Br hydrogen bonds and weak C—H···O and C—H···Br interactions (Table1) consolidate the crystal packing, which exhibits relatively short intermolecular Br···Br contacts of 3.429 (3) Å.

### Experimental

The reaction was carried out by the solvothermal method. Bromoacetic acid (0.138 g,1 mmol) and cupric acetate(0.199 g, 1 mmol) and 2,2'-bipyridine(0.312 g, 2 mmol) were added to the airtight vessel with 1:2 ratio of ethanol and water. The resulting blue solution was filtered, and blue block-shaped crystals were obtained after several days. Yield 81%. Elemental analysis: calcd. for C_22_H_20_Br_3_Cu_1_N_4_O_2.5_: C 38.65, H 2.95, N 8.19; found: C 38.45, H 2.79, N 8.22. The elemental analyses were performed with PERKIN ELMER MODEL 2400 SERIES II.

### Refinement

All H atoms were geometrically positioned (C—H 0.93-0.97 Å, O—H 0.82-0.85 Å), abd refined as riding, with *U*_iso_(H) = 1.2-1.5 *U*_eq_(C, O).

### Crystal data

**Table:**

[CuBr(C_10_H_8_N_2_)_2_]Br·C_2_H_3_BrO_2_·0.5H_2_O	*Z* = 2
*M**_r_* = 683.69	*F*(000) = 668
Triclinic, *P*1	*D*_x_ = 1.809 Mg m^−^^3^
*a* = 8.580 (4) Å	Mo *K*α radiation, λ = 0.71073 Å
*b* = 12.125 (6) Å	Cell parameters from 1942 reflections
*c* = 13.212 (6) Å	θ = 2.5–26.3°
α = 70.295 (9)°	µ = 5.67 mm^−^^1^
β = 81.427 (8)°	*T* = 273 K
γ = 76.669 (9)°	Block, blue
*V* = 1255.3 (11) Å^3^	0.30 × 0.27 × 0.26 mm

### Data collection

**Table:**

Bruker SMART APEX diffractometer	4384 independent reflections
Radiation source: fine-focus sealed tube	2689 reflections with *I* > 2σ(*I*)
graphite	*R*_int_ = 0.113
phi and ω scans	θ_max_ = 25.1°, θ_min_ = 1.8°
Absorption correction: multi-scan (*SADABS*; Bruker, 2005)	*h* = −8→10
*T*_min_ = 0.281, *T*_max_ = 0.320	*k* = −11→14
6360 measured reflections	*l* = −15→15

### Refinement

**Table:**

Refinement on *F*^2^	Primary atom site location: structure-invariant direct methods
Least-squares matrix: full	Secondary atom site location: difference Fourier map
*R*[*F*^2^ > 2σ(*F*^2^)] = 0.067	Hydrogen site location: inferred from neighbouring sites
*wR*(*F*^2^) = 0.183	H-atom parameters constrained
*S* = 1.00	*w* = 1/[σ^2^(*F*_o_^2^) + (0.093*P*)^2^] where *P* = (*F*_o_^2^ + 2*F*_c_^2^)/3
4384 reflections	(Δ/σ)_max_ < 0.001
299 parameters	Δρ_max_ = 1.20 e Å^−^^3^
3 restraints	Δρ_min_ = −1.10 e Å^−^^3^

### Special details

**Table:**

Geometry. All e.s.d.'s (except the e.s.d. in the dihedral angle between two l.s. planes) are estimated using the full covariance matrix. The cell e.s.d.'s are taken into account individually in the estimation of e.s.d.'s in distances, angles and torsion angles; correlations between e.s.d.'s in cell parameters are only used when they are defined by crystal symmetry. An approximate (isotropic) treatment of cell e.s.d.'s is used for estimating e.s.d.'s involving l.s. planes.
Refinement. Refinement of *F*^2^ against ALL reflections. The weighted *R*-factor *wR* and goodness of fit *S* are based on *F*^2^, conventional *R*-factors *R* are based on *F*, with *F* set to zero for negative *F*^2^. The threshold expression of *F*^2^ > σ(*F*^2^) is used only for calculating *R*-factors(gt) *etc*. and is not relevant to the choice of reflections for refinement. *R*-factors based on *F*^2^ are statistically about twice as large as those based on *F*, and *R*- factors based on ALL data will be even larger.

### Fractional atomic coordinates and isotropic or equivalent isotropic displacement parameters (Å^2^)

**Table:**

	*x*	*y*	*z*	*U*_iso_*/*U*_eq_	Occ. (<1)
Cu1	0.29541 (12)	0.96050 (9)	0.26233 (8)	0.0355 (3)	
N1	0.2936 (8)	1.0281 (6)	0.3915 (5)	0.0343 (17)	
N2	0.1969 (9)	0.8397 (6)	0.3826 (5)	0.0396 (18)	
N3	0.4085 (8)	1.0763 (6)	0.1487 (5)	0.0357 (17)	
N4	0.5077 (8)	0.8468 (6)	0.2359 (5)	0.0335 (17)	
O1	0.5681 (9)	0.4552 (7)	0.1253 (6)	0.072 (2)	
H1	0.4898	0.4612	0.1684	0.108*	
O2	0.6357 (9)	0.2741 (8)	0.2416 (6)	0.090 (3)	
O3	0.4997 (16)	0.4203 (11)	0.4374 (10)	0.057 (4)	0.50
H3A	0.4339	0.4667	0.3920	0.068*	0.50
H3B	0.5632	0.3711	0.4099	0.068*	0.50
Br1	0.96870 (13)	0.40992 (10)	0.14479 (8)	0.0541 (3)	
Br2	0.05033 (11)	1.02044 (9)	0.16420 (7)	0.0468 (3)	
Br3	0.23105 (12)	0.51574 (10)	0.24364 (9)	0.0574 (3)	
C1	0.6651 (12)	0.3530 (9)	0.1639 (8)	0.050 (3)	
C2	0.8186 (12)	0.3421 (10)	0.0945 (8)	0.054 (3)	
H2A	0.8002	0.3855	0.0195	0.064*	
H2B	0.8624	0.2589	0.1010	0.064*	
C3	0.3481 (11)	1.1248 (8)	0.3882 (8)	0.046 (2)	
H3	0.3908	1.1706	0.3228	0.055*	
C4	0.3426 (13)	1.1581 (10)	0.4791 (10)	0.063 (3)	
H4	0.3845	1.2235	0.4762	0.075*	
C5	0.2718 (12)	1.0902 (10)	0.5760 (8)	0.053 (3)	
H5	0.2599	1.1127	0.6379	0.063*	
C6	0.2192 (11)	0.9878 (9)	0.5783 (7)	0.044 (2)	
H6	0.1776	0.9388	0.6423	0.053*	
C7	0.2305 (9)	0.9621 (8)	0.4850 (7)	0.032 (2)	
C8	0.1752 (10)	0.8567 (8)	0.4793 (6)	0.037 (2)	
C9	0.1025 (11)	0.7787 (8)	0.5660 (7)	0.044 (2)	
H9	0.0885	0.7895	0.6334	0.053*	
C10	0.0516 (13)	0.6868 (10)	0.5531 (8)	0.060 (3)	
H10	0.0025	0.6345	0.6109	0.072*	
C11	0.0743 (13)	0.6724 (10)	0.4521 (8)	0.060 (3)	
H11	0.0404	0.6102	0.4408	0.072*	
C12	0.1468 (13)	0.7503 (9)	0.3701 (8)	0.056 (3)	
H12	0.1618	0.7404	0.3022	0.067*	
C13	0.3448 (12)	1.1931 (8)	0.1070 (8)	0.051 (3)	
H13	0.2437	1.2231	0.1343	0.061*	
C14	0.4260 (13)	1.2703 (9)	0.0240 (8)	0.053 (3)	
H14	0.3792	1.3504	−0.0047	0.063*	
C15	0.5761 (12)	1.2258 (9)	−0.0145 (7)	0.046 (2)	
H15	0.6328	1.2757	−0.0695	0.055*	
C16	0.6409 (11)	1.1099 (9)	0.0276 (7)	0.047 (2)	
H16	0.7429	1.0799	0.0014	0.056*	
C17	0.5572 (10)	1.0337 (8)	0.1101 (6)	0.034 (2)	
C18	0.6133 (10)	0.9048 (8)	0.1607 (6)	0.035 (2)	
C19	0.7675 (11)	0.8444 (9)	0.1358 (8)	0.049 (3)	
H19	0.8377	0.8852	0.0837	0.059*	
C20	0.8125 (13)	0.7270 (10)	0.1879 (9)	0.060 (3)	
H20	0.9151	0.6865	0.1735	0.072*	
C21	0.7010 (13)	0.6653 (9)	0.2656 (9)	0.059 (3)	
H21	0.7287	0.5839	0.3015	0.071*	
C22	0.5528 (12)	0.7292 (9)	0.2856 (8)	0.053 (3)	
H22	0.4799	0.6893	0.3359	0.063*	

### Atomic displacement parameters (Å^2^)

**Table:**

	*U*^11^	*U*^22^	*U*^33^	*U*^12^	*U*^13^	*U*^23^
Cu1	0.0381 (6)	0.0378 (6)	0.0242 (6)	−0.0070 (5)	0.0117 (4)	−0.0077 (5)
N1	0.033 (4)	0.043 (4)	0.028 (4)	−0.007 (3)	0.008 (3)	−0.016 (4)
N2	0.047 (5)	0.040 (4)	0.028 (4)	−0.016 (4)	0.013 (3)	−0.007 (3)
N3	0.033 (4)	0.036 (4)	0.027 (4)	−0.004 (3)	0.009 (3)	−0.002 (3)
N4	0.037 (4)	0.033 (4)	0.024 (4)	0.000 (3)	0.000 (3)	−0.007 (3)
O1	0.059 (5)	0.053 (5)	0.069 (5)	0.006 (4)	0.012 (4)	0.007 (4)
O2	0.065 (6)	0.085 (6)	0.065 (5)	−0.006 (5)	0.008 (4)	0.034 (5)
O3	0.080 (10)	0.041 (8)	0.048 (8)	−0.015 (7)	−0.047 (8)	0.007 (6)
Br1	0.0528 (7)	0.0612 (7)	0.0445 (6)	−0.0112 (5)	0.0059 (5)	−0.0156 (5)
Br2	0.0376 (6)	0.0617 (7)	0.0353 (5)	−0.0075 (5)	0.0033 (4)	−0.0117 (5)
Br3	0.0428 (6)	0.0652 (7)	0.0613 (7)	−0.0074 (5)	0.0032 (5)	−0.0212 (6)
C1	0.049 (6)	0.050 (6)	0.046 (6)	−0.007 (5)	−0.007 (5)	−0.008 (5)
C2	0.049 (6)	0.060 (7)	0.044 (6)	−0.002 (5)	0.005 (5)	−0.014 (5)
C3	0.052 (6)	0.042 (6)	0.042 (6)	−0.010 (5)	0.006 (5)	−0.014 (5)
C4	0.063 (8)	0.064 (7)	0.083 (9)	−0.008 (6)	−0.024 (6)	−0.046 (7)
C5	0.057 (7)	0.069 (7)	0.036 (6)	0.004 (6)	−0.012 (5)	−0.028 (6)
C6	0.044 (6)	0.055 (6)	0.023 (5)	0.008 (5)	−0.002 (4)	−0.013 (5)
C7	0.021 (4)	0.039 (5)	0.029 (5)	0.008 (4)	−0.006 (3)	−0.011 (4)
C8	0.031 (5)	0.046 (6)	0.021 (5)	−0.003 (4)	0.008 (4)	0.000 (4)
C9	0.042 (6)	0.050 (6)	0.025 (5)	−0.008 (5)	0.004 (4)	0.003 (4)
C10	0.064 (7)	0.071 (8)	0.037 (6)	−0.033 (6)	0.012 (5)	0.000 (5)
C11	0.077 (8)	0.060 (7)	0.043 (6)	−0.033 (6)	0.014 (5)	−0.012 (5)
C12	0.077 (8)	0.053 (7)	0.037 (6)	−0.031 (6)	0.009 (5)	−0.009 (5)
C13	0.045 (6)	0.043 (6)	0.051 (6)	−0.002 (5)	0.005 (5)	−0.007 (5)
C14	0.065 (7)	0.035 (5)	0.048 (6)	−0.012 (5)	−0.012 (5)	0.004 (5)
C15	0.062 (7)	0.048 (6)	0.028 (5)	−0.021 (5)	0.001 (4)	−0.006 (5)
C16	0.045 (6)	0.062 (7)	0.034 (5)	−0.023 (5)	0.014 (4)	−0.016 (5)
C17	0.039 (5)	0.043 (5)	0.020 (4)	−0.012 (4)	−0.002 (4)	−0.010 (4)
C18	0.039 (5)	0.047 (6)	0.022 (4)	−0.007 (4)	−0.003 (4)	−0.014 (4)
C19	0.030 (5)	0.062 (7)	0.059 (7)	−0.004 (5)	0.006 (4)	−0.031 (6)
C20	0.051 (7)	0.061 (8)	0.068 (7)	0.010 (6)	−0.006 (6)	−0.033 (6)
C21	0.067 (8)	0.043 (6)	0.060 (7)	0.012 (5)	−0.022 (6)	−0.014 (6)
C22	0.044 (6)	0.060 (7)	0.041 (6)	0.003 (5)	−0.003 (5)	−0.007 (5)

### Geometric parameters (Å, °)

**Table:**

Cu1—N3	1.985 (6)	C6—C7	1.355 (12)
Cu1—N2	1.997 (7)	C6—H6	0.9300
Cu1—N4	2.078 (7)	C7—C8	1.489 (13)
Cu1—N1	2.125 (7)	C8—C9	1.388 (11)
Cu1—Br2	2.471 (2)	C9—C10	1.355 (14)
N1—C7	1.340 (10)	C9—H9	0.9300
N1—C3	1.344 (11)	C10—C11	1.382 (14)
N2—C12	1.321 (12)	C10—H10	0.9300
N2—C8	1.341 (11)	C11—C12	1.354 (13)
N3—C13	1.347 (11)	C11—H11	0.9300
N3—C17	1.354 (10)	C12—H12	0.9300
N4—C22	1.343 (12)	C13—C14	1.395 (13)
N4—C18	1.362 (10)	C13—H13	0.9300
O1—C1	1.309 (12)	C14—C15	1.370 (14)
O1—H1	0.8200	C14—H14	0.9300
O2—C1	1.185 (11)	C15—C16	1.341 (14)
O3—H3A	0.8500	C15—H15	0.9300
O3—H3B	0.8500	C16—C17	1.397 (12)
Br1—C2	1.972 (11)	C16—H16	0.9300
C1—C2	1.496 (13)	C17—C18	1.472 (12)
C2—H2A	0.9700	C18—C19	1.408 (12)
C2—H2B	0.9700	C19—C20	1.347 (14)
C3—C4	1.381 (13)	C19—H19	0.9300
C3—H3	0.9300	C20—C21	1.431 (15)
C4—C5	1.406 (15)	C20—H20	0.9300
C4—H4	0.9300	C21—C22	1.368 (14)
C5—C6	1.406 (14)	C21—H21	0.9300
C5—H5	0.9300	C22—H22	0.9300
			
N3—Cu1—N2	175.6 (3)	C6—C7—C8	122.2 (8)
N3—Cu1—N4	80.1 (3)	N2—C8—C9	119.5 (9)
N2—Cu1—N4	97.1 (3)	N2—C8—C7	116.3 (7)
N3—Cu1—N1	98.2 (3)	C9—C8—C7	124.2 (8)
N2—Cu1—N1	79.9 (3)	C10—C9—C8	120.4 (9)
N4—Cu1—N1	114.5 (3)	C10—C9—H9	119.8
N3—Cu1—Br2	93.4 (2)	C8—C9—H9	119.8
N2—Cu1—Br2	91.0 (2)	C9—C10—C11	118.7 (10)
N4—Cu1—Br2	127.57 (19)	C9—C10—H10	120.7
N1—Cu1—Br2	117.90 (19)	C11—C10—H10	120.7
C7—N1—C3	119.6 (8)	C12—C11—C10	118.8 (10)
C7—N1—Cu1	112.7 (5)	C12—C11—H11	120.6
C3—N1—Cu1	127.7 (6)	C10—C11—H11	120.6
C12—N2—C8	120.0 (8)	N2—C12—C11	122.6 (10)
C12—N2—Cu1	123.8 (6)	N2—C12—H12	118.7
C8—N2—Cu1	116.2 (6)	C11—C12—H12	118.7
C13—N3—C17	118.7 (7)	N3—C13—C14	121.9 (9)
C13—N3—Cu1	123.9 (6)	N3—C13—H13	119.0
C17—N3—Cu1	117.4 (5)	C14—C13—H13	119.0
C22—N4—C18	118.6 (8)	C15—C14—C13	118.7 (9)
C22—N4—Cu1	128.6 (6)	C15—C14—H14	120.7
C18—N4—Cu1	112.8 (6)	C13—C14—H14	120.7
C1—O1—H1	109.5	C16—C15—C14	119.7 (9)
H3A—O3—H3B	109.7	C16—C15—H15	120.2
O2—C1—O1	125.6 (10)	C14—C15—H15	120.2
O2—C1—C2	122.2 (10)	C15—C16—C17	120.8 (9)
O1—C1—C2	112.1 (9)	C15—C16—H16	119.6
C1—C2—Br1	106.9 (7)	C17—C16—H16	119.6
C1—C2—H2A	110.3	N3—C17—C16	120.2 (8)
Br1—C2—H2A	110.3	N3—C17—C18	113.9 (7)
C1—C2—H2B	110.3	C16—C17—C18	126.0 (8)
Br1—C2—H2B	110.3	N4—C18—C19	121.4 (8)
H2A—C2—H2B	108.6	N4—C18—C17	115.8 (7)
N1—C3—C4	122.0 (9)	C19—C18—C17	122.7 (8)
N1—C3—H3	119.0	C20—C19—C18	119.4 (10)
C4—C3—H3	119.0	C20—C19—H19	120.3
C3—C4—C5	117.9 (10)	C18—C19—H19	120.3
C3—C4—H4	121.0	C19—C20—C21	119.3 (10)
C5—C4—H4	121.0	C19—C20—H20	120.3
C4—C5—C6	119.1 (9)	C21—C20—H20	120.3
C4—C5—H5	120.5	C22—C21—C20	118.3 (10)
C6—C5—H5	120.5	C22—C21—H21	120.9
C7—C6—C5	118.5 (9)	C20—C21—H21	120.9
C7—C6—H6	120.8	N4—C22—C21	123.0 (10)
C5—C6—H6	120.8	N4—C22—H22	118.5
N1—C7—C6	122.8 (8)	C21—C22—H22	118.5
N1—C7—C8	114.9 (7)		

### Hydrogen-bond geometry (Å, °)

**Table:**

*D*—H···*A*	*D*—H	H···*A*	*D*···*A*	*D*—H···*A*
O1—H1···Br3	0.82	2.34	3.129 (7)	163
O3—H3A···Br3	0.85	2.64	3.426 (11)	154
C14—H14···O1^i^	0.93	2.46	3.254 (12)	144
C16—H16···Br2^i^	0.93	3.01	3.835 (9)	149
C3—H3···O2^ii^	0.93	2.60	3.374 (12)	141
C4—H4···O3^ii^	0.93	2.65	3.578 (16)	172
C10—H10···Br1^iii^	0.93	3.13	3.751 (10)	126
C2—H2B···Br2^iv^	0.97	2.88	3.815 (11)	163
C13—H13···Br1^v^	0.93	2.89	3.750 (10)	154

Symmetry codes: (i) −*x*+1, −*y*+2, −*z*; (ii) *x*, *y*+1, *z*; (iii) −*x*+1, −*y*+1, −*z*+1; (iv) *x*+1, *y*−1, *z*; (v) *x*−1, *y*+1, *z*.
